# Clinical findings and treatment in cattle with caecal dilatation

**DOI:** 10.1186/1746-6148-8-75

**Published:** 2012-07-09

**Authors:** Ueli Braun, Christine Beckmann, Christian Gerspach, Michael Hässig, Evelyne Muggli, Gabriela Knubben-Schweizer, Karl Nuss

**Affiliations:** 1 Department of Farm Animals, Vetsuisse Faculty, University of Zurich, Zurich, Switzerland

## Abstract

**Background:**

This retrospective study describes the clinical and laboratory findings, treatment and outcome of 461 cattle with caecal dilatation.

**Results:**

The general condition and demeanor were abnormal in 93.1% of cases, and 32.1% of the patients had colic. Ruminal motility was reduced or absent in 78.3% of cattle. In 82.6% of cases, swinging and/or percussion auscultation were positive on the right side, and 82.4% had little or no faeces in the rectum. Caecal dilatation could be diagnosed via rectal palpation in 405 (88.0%) cattle. There was caudal displacement of the dilated caecum in 291 patients, torsion around the longitudinal axis in 20 and retroflexion in 94. The most important laboratory finding was hypocalcaemia, which occurred in 85.1% of cases. Of the 461 cattle, 122 (26.5%) initially received conservative therapy (intravenous fluids, neostigmine, calcium borogluconate) and 329 (71.4%) underwent surgical treatment. Ten patients were slaughtered or euthanased after the initial physical examination. Of the 122 cattle that received conservative treatment, 42 did not respond after one to two days of therapy and required surgical treatment. The final number of cattle that were operated was 371 (80.5%). Because of a grave prognosis, 24 cases were euthanased or slaughtered intraoperatively. Another 24 cattle did not respond to one or more operations and were euthanased or slaughtered. Of the 461 patients, 403 (87.4%) responded to either conservative or surgical treatment and were cured, and 58 were euthanased or slaughtered.

**Conclusions:**

Caecal dilatation can usually be diagnosed based on clinical findings and treated conservatively or surgically. Swinging and percussion auscultation as well as rectal examination are important diagnostic tools. Conservative treatment is not rewarding in cattle considered surgical candidates with suspected caecal torsion or retroflexion and surgery should not be delayed in these patients.

## Background

In cattle with caecal dilatation, there is distension of the caecum, which may be accompanied by displacement, torsion or retroflexion of the organ and additional distension of the spiral colon [1]. With distension alone, the apex of the caecum is displaced toward or into the pelvic inlet. With torsion, the distended caecum rotates about its longitudinal axis, and with retroflexion, the caecum folds dorsally or ventrally in the ileocaecal region, resulting in a cranial orientation of the apex. Caecal dilatation is associated with partial or complete cessation of the passage of intestinal contents. Studies in cattle that were treated surgically for caecal dilatation showed that the position of the displaced caecum varied greatly and was not restricted to the previous description [2,3]. Older reports of caecal dilatation, usually involving only a few cases, have been summarised [4-7]. In the past 25 years, the clinical findings of caecal dilatation in cattle have been described by many authors [4,8,9]. The most important diagnostic tool is rectal examination, which can be used to palpate dilatation, displacement and sometimes torsion of the caecum in 95% of cases [4]. In cases where the caecum cannot be palpated transrectally, there is usually marked retroflexion of the organ, which can be detected via ultrasonography or laparotomy [10]. The results of haematological and blood biochemical analyses are not diagnostic for caecal dilatation but serve to estimate the severity of disease. For example, an elevation in the haematocrit may indicate dehydration, and increases in the concentration of blood urea nitrogen may be a sign of prerenal azotemia. A previous study of caecal dilatation [5] reported disturbances in electrolyte concentrations, including hypocalcaemia (61% of cases), hypophosphataemia (35%) and hypermagnesaemia (42%). For epidemiological reasons, it is interesting to note that certain feeds are associated with caecal dilatation [11]. Corn silage and mineral supplements decrease the risk of caecal dilatation, whereas lack of salt increases the risk. A detailed overview of treatment options has been described [7]. Generally, treatment consists of either conservative therapy using parasympathomimetic drugs such as neostigmine [6] or bethanechol [12] or surgical emptying of the caecum. Amputation of the caecum should be reserved for an atonic or severely diseased caecum or recurrent caecal dilatation. Previous studies of bovine caecal dilatation have used a wide range of sample size: 30 [10], 40 [12], 80 [7], 84 [9], 111 [4], 158 [11] and 203 cattle [8]. The goal of the present retrospective paper was to investigate the clinical and laboratory findings and treatment in 461 cattle with caecal dilatation.

## Methods

### Animals

A total of 461 cattle with caecal dilatation were admitted to the Department of Farm Animals, University of Zürich, from June 25, 1997 to June 28, 2008. With the exception of two patients, all the cattle were female. There were 261 Swiss Braunvieh, 137 Simmental, 54 Holstein-Friesian and nine crossbred cattle, which ranged in age from 0.25 to 12.5 years (Mean ± sd = 4.7 ± 1.9 years, Median 4.5 years).

### Clinical examination

All cattle underwent a thorough clinical examination as described [13]. The general condition and demeanour, rectal temperature, heart rate, respiratory rate and lung sounds were determined. Swinging and/or percussion ausculatation on both sides of the abdomen, tests for a reticular foreign body and rectal palpation were also carried out. Urine was examined using a urine test strip (Combur9-Test®, Roche, Basel).

### Laboratory examinations

Haematological analysis included the determination of PCV, total leukocyte count, and the concentrations of fibrinogen and total protein in EDTA blood samples using an automated blood analyzer (CELL-Dyn 3500, Abbott Diagnostics Division, Baar). The concentrations of serum bilirubin, urea nitrogen, sodium, chloride, potassium, calcium, inorganic phosphorus and magnesium was determined at 37°C using an automated analyser (Cobas-Integra-800-Analyser, Roche Diagnostics, Basel) and the manufacturer’s reagents (Roche-Reagents) according to the International Federation of Clinical Chemistry and Laboratory Medicine (IFCC). Venous blood gas analysis was done using an automated analyser (RapidLab 248, (Siemens Schweiz AG, Zürich). The concentration of chloride in rumen fluid obtained via a stomach tube was carried out in 404 cows using an MK-II-Chloride Analyser 9265 (Sherwood, Cambridge).

### Ultrasonographic examination of the abdomen

In 56 cows in which a suspected caecal dilatation could not be diagnosed via rectal palpation, abdominal ultrasonography was done as described previously [10] to aid in diagnosis.

### Treatment

Conservative treatment was instituted when the general condition and demeanour of the patient were normal or only mildly abnormal, faecal output was normal or mildly reduced and the caecum was dilated and displaced caudally with no torsion. Cattle treated conservatively received 10 litres sodium chloride and glucose solution (50 g glucose and 9 g sodium chloride/l) daily administered via an indwelling jugular vein catheter (Abbocath-T 14 G, length 14 cm, diameter 2 mm; Abbott AG, Baar). In addition, 5 litres sodium chloride and glucose solution containing 42.5 mg neostigmine (Konstigmin®, Vétoquinol AG, Ittigen, Switzerland), and 500 ml of a 40% calcium borogluconate solution with 6% magnesium hypophosphite (Calcamyl-40MP®, Graeub AG, Bern, Switzerland) were administered as an intravenous drip. Cattle with signs of visceral pain were given 30 mg/kg metamizole (Vetalgin®, Veterinaria AG, Pfäffikon Switzerland). Electrolyte deficiencies were corrected as follows: Cows received calcium borogluconate intravenously for hypocalcaemia, magnesium hypophosphite intravenously for hypomagnesaemia (see above), sodium chloride and glucose solution intravenously for hyponatraemia and hypochloraemia (see above), potassium chloride per os for hypokalaemia and sodium dihydrogen phosphate per os for hypophosphataemia. When the heart rate decreased to less than 60 bpm, the neostigmine infusion was temporarily discontinued. The cattle were fasted for 48 hours, although a small amount of hay was offered daily to evaluate appetite. A positive finding was recorded when the patient immediately ate the hay.

Surgical treatment was carried out in cattle that had a poor general condition and demeanour, colic, absence of faeces, caecal torsion or retroflexion of the caecum. Operations were done on standing cattle using a proximal paravertebral local anaesthesia. A right flank laparotomy allowed exteriorization of the dilated caecum. An incision approximately 4 to 5 cm long was made in the apex of the caecum and the contents were emptied into a bucket. The remainder of the caecum, the proximal loop of the ascending colon and, if possible, the obstipated parts of the spiral colon were also emptied via gentle massage. The caecal incision was closed using monofilament absorbable suture material (Biosyn 2–0, Tyco Healthcare Switzerland AG, Wollerau) in a double Cushing suture pattern. The incisional area was thoroughly lavaged with lukewarm physiological saline solution and diluted iodine solution. After evaluating the vitality of the caecum by administering drops of neostigmine solution onto the organ, it was replaced into the abdomen. Before closing the laparotomy incision, the position and degree of filling of the caecum were assessed. When the caecum rapidly filled with ingesta, it was incised and emptied a second time. When there was absence of caecal contractions, necrotic regions in the caecal wall or recurrence of the dilatation following a previous surgery, partial amputation of the organ was carried out. The caecum was anaesthetised by blocking the ileocaecal nerve, and the organ was amputated as close to the ileocaecal junction as possible. The antimesenteric ileal branches of the caecal artery and vein were ligated, and the ileocaecal fold close to the ileal junction was severed. Then two large intestinal clamps were placed on the base of the caecum, and the organ was amputated by cutting between the clamps. The caecal stump was closed using absorbable suture material (Biosyn 2–0, Tyco Healthcare Switzerland AG) in a double Cushing suture pattern. The abdominal cavity was rinsed with 1 litre physiological saline solution (Provet AG, Lyssach) containing 4 x 10^6^ IU benzyl penicillin and 1 g neomycin (NPS®, Veterinaria AG). The peritoneum and abdominal wall were closed in four layers using absorbable suture material (Polysorb 2, Polysorb 0, Tyco Healthcare Switzerland AG) in a continuous suture pattern. The skin incision was closed with staples (Appose ULC, 35 W, Tyco Healthcare Switzerland AG). Postoperatively, the cattle received 15‘000 IU/kg benzyl penicillin (Procacillin®, Veterinaria AG) administered intramuscularly once daily and 1 mg/kg flunixin meglumine (Finadyne®, Veterinaria AG) administered intravenously once daily for three days. Treatment with sodium chloride and glucose solution, neostigmine, calcium borogluconate, metamizole and electrolytes as well as fasting was carried out similar to the cows treated conservatively.

### Clinical outcome

The heart and respiratory rates, rectal temperature, ruminal and intestinal motility, faecal output and consistency, swinging and percussion auscultation and rectal examination were carried out once daily in all the patients. In the first 48 hours after the start of treatment, the general condition and demeanor, heart rate, respiratory rate and faecal output were assessed every hour so that the neostigmine infusion could be slowed or discontinued if colic or bradycardia occurred. The number of days required for the general condition and faecal output of the patients to normalise as well as the number of days patients remained in the clinic were recorded.

### Statistical analysis

The program StatView 5.1 (SAS Institute Inc., Cary, NC) was used for statistical calculations. Normally distribution was tested by Wilk-Shapiro test. Frequencies, means and standard deviations were calculated and differences were analysed using an unpaired *t*-test for normally distributed variables. Frequencies, median and range were calculated for not normally distributed variables. The differences between not normal distributed variables were tested by ANOVA and Kruskal-Wallis test for equality of populations. The frequency distributions of clinical evaluations (rumen motility, foreign body tests, swinging and percussion auscultation on the right side, rectal findings) of cows with successful and unsuccessful outcomes were compared using a chi-square test, the means and medians of continuous variables of clinical, haematological and biochemical evaluations of cows with successful and unsuccessful outcomes were compared using a *t*-test and theWilcoxon rank sum test for not normally distributed variables. A value of P < 0.05 was considered significant.

## Results

### Clinical findings

The general condition and demeanour were abnormal in 429 (93.1%) cattle: changes were mild in 194, moderate in 202 and severe in 33 patients. Signs of colic which included, shifting of weight from one hind foot to the other, restlessness, tail thrashing, sinking of the back and/or kicking were seen in 148 (32.1%) cattle. Forceful kicking, sweating, frequent lying down and standing up were seen occasionally. Twenty patients had bilateral enlargement of the abdomen, seven had a pear-shaped abdomen and 22 had slight distention of the right flank.

The heart rate varied from 42 to 160 bpm (mean ± sd = 75.2 ± 14.3 bpm) and was normal at 60 to 80 bpm in 322 (69.8%) (Table 1). Bradycardia (42–59 bpm) occurred in 26 cattle and 113 had tachycardia (81–160 bpm). Tachycardia was mild (81–110 bpm) in 99 cases and severe (101–160 bpm) in 14. There was no significant difference among the average heart rate of cows with caecal dilatation (74.2 bpm), caecal torsion (74.4 bpm) and caecal retroflexion (77.2 bpm).

**Table 1:** Clinical findings in 461 cows with caecal dilation

Variable	Finding	Number of cows
(Mean ± sd; Median, minimum, maximum)		
[P value from Wilk-Shapiro test]		
Heart rate (n = 461)	Normal (60 – 80)	322
(72, 42, 160 bpm)	Bradycardia (42 – 59)	26
[0.001]	Tachycardia (81 – 160)	113
Respiratory rate (n = 461)	Normal (15 – 25)	254
(24, 12, 108 breaths per minute	Decreased (12 – 14)	6
[0.000]	Increased (26 – 108)	201
Rectal temperature (n = 457)	Normal (38.5 – 39.0)	229
(38.7 ± 0.6 °C)	Decreased (36.5 – 38.5)	115
[0.936]	Increased (39.1 – 40.4)	113
	Not measurable (pneumorectum)	4
Rumen motility (n = 461)	Normal	87
	Decreased	260
	Absent	101
	Increased	13
Foreign body tests (n = 445)	All tests negative	354
	Back grip positive^1^	42
	Pole test positive^1^	19
	Percussion of the reticulum positive^1^	7
	Two or three tests positive	23
Swinging and percussion auscultation on the right side (n = 461)	Both negative (normal)	80
	Both positive	272
	Only swinging auscultation positive	72
	Only percussion auscultation positive	37
Rectal findings (n = 461)	Caudal displacement of the caecum	291
	Torsion of the caecum	20
	Retroflexion of the caecum	94
	Caecum not palpable	56

^1^ Elicited a grunt in at least 3 of 4 tests.

The respiratory rate ranged from 12 to 108 breaths per minute (median, minimum, maximum = 24, 12, 108 breaths per minute) and was normal at 15 to 25 breaths per minute in 254 (55.1%) cattle. The respiratory rate was lower than normal (12–14 breaths per minute) in six cases and higher than normal (26–108 breaths per minute) in 201. A mild increase in the respiratory rate (26–35 breaths per minute) was seen in 124 cattle, a moderate increase (36 - 45 breaths per minute) in 61 and a severe increase (46–108 breaths per minute) in 16.

The rectal temperature ranged from 35.7 to 40.4 °C (38.7 ± 0.6 °C) and was normal (38.5 - 39.0 °C) in 229 (50.1%) cases. There was a mild increase in rectal temperature (39.1 - 39.5 °C) in 89 cattle and a marked increase (39.6 - 40.4 °C) in 24. A lower than normal rectal temperature (< 38.5 °C) was recorded in 115 (25.2%) cases. The rectal temperature could not be measured in four animals because of pneumorectum.

Ruminal motility was reduced (n = 260) or absent (n = 101) in 361 (78.3%) cattle. In 87 cases it was normal and in 13 cases increased.

In 91 (20.4%) cattle, one or more tests for a reticular foreign body were positive. A total of 68 cattle had one positive test: the withers pinch was positive in 42 cattle, the pole test was positive in 19 and percussion of the reticulum was positive in seven. In 23 cases, a minimum of two or three tests were positive. Testing could not be carried out in 16 patients because they did not cooperate.

Swinging and/or percussion auscultation on the right side of the abdomen were positive in 381 (82.6%) cattle; both tests were positive in 272 cases, swinging auscultation alone was positive in 72 and percussion auscultation alone was positive in 37. Both tests were negative on the left side of all the cattle.

Rectal findings in 380 (82.4%) cattle included scant (n = 274) or no faeces (n = 106) and blood, mucus or fibrin (n = 121).

Caecal dilatation could be diagnosed via rectal palpation in 405 (87.9%) cattle. There was caudal displacement of the dilated caecum in 291 patients, torsion around the longitudinal axis in 20 and retroflexion in 94. Of the latter, the caecum was located transversely cranial to the pelvic inlet in 22. Caecal dilatation could not be palpated transrectally in 56 (12.1%) cattle; four of these had dilated loops of small intestine and two had dilation of the spiral colon, all of which were palpated transrectally.

### Laboratory findings

The most common haematological abnormalities were a higher than normal concentration of total protein in 39.8%, leukocytosis 38.8% and a lower than normal PCV in 24.6% of cattle (Table 2). The concentration of fibrinogen was higher than normal in 21.4% of cattle.

**Table 2:** Haematological, biochemical and venous blood gas analyses in cows with caecal dilatation

Variable	Finding	Number of cows
(Mean ± sd, median, minimum, maximum)		
[P value from Wilk-Shapiro test]		
Haematological variables		
PCV (n = 456)	Normal (30 – 35%)	240
(33.0 ± 5.0%)	Decreased (11 – 29%)	112
[0.783]	Increased (36 – 58%)	104
Total leukocyte count (n = 456)	Normal (5000 – 10’000/μl)	268
(9450, 1000, 35‘100/μl)	Decreased (1000 - 4999/μl)	11
[0.000]	Increased (10’001 – 35‘100/μl)	177
Total protein (n = 455)	Normal (60 – 80 g/l)	270
(78.7 ± 8.2 g/l)	Decreased (32 – 59 g/l)	4
[0.962]	Increased (81 – 114 g/l)	181
Fibrinogen (n = 454)	Normal (4 – 7 g/l)	323
(5.9 ± 2.1 g/l)	Decreased (2 – 3 g/l)	34
[0.185]	Increased (8 – 14 g/l)	97
Biochemical variables		
Bilirubin (n = 454)	Normal (1.5 – 6.5 μmol/l)	303
(5.4, 0.6, 29.1 μmol/l)	Decreased (0.6 – 1.4 μmol/l)	5
[0.000]	Increased (6.6 – 29.1 μmol/l)	146
Urea (n = 459)	Normal (2.4 – 6.5 mmol/l)	264
(6.0, 1.4, 33.3 mmol/l)	Decreased (1.4 – 2.3)	8
[0.003]	Increased (6.6 – 33.9 mmol/l)	118
Calcium (n = 348)	Normal (2.3 – 2.6 mmol/l)	49
(2.1 ± 0.3 mmol/l)	Decreased (1.0 – 2.2 mmol/l)	296
[0.619]	Increased (2.7 – 3.6 mmol/l)	3
Inorganic phosphorus (n = 346)	Normal (1.3 – 2.4 mmol/l)	188
(1.5 ± 0.6 mmol/l)	Decreased (0.3 – 1.2 mmol/l)	140
[0.000]	Increased (2.5 – 4.5 mmol/l)	18
Magnesium (n = 347)	Normal (0.8 – 1.0 mmol/l)	186
(1.0 ± 0.2 mmol/l)	Decreased (0.6 – 0.7)	36
[0.760]	Increased (1.1 – 2.3 mmol/l)	125
Sodium (n = 459)	Normal (145 – 155 mmol/l)	266
(144.6 ± 3.7 mmol/l)	Decreased (129 – 144 mmol/l)	191
[0.184]	Increased (156 – 160 mmol/l)	2
Chloride (n = 458)	Normal (96 – 105 mmol/l)	313
(101, 68, 119 mmol/l)	Decreased (57 – 95 mmol/l)	59
[0.001]	Increased (106 – 119 mmol/l)	86
Potassium (n = 456)	Normal (4.0 – 5.0 mmol/l)	175
(3.9 ± 0.7 mmol/l)	Decreased (1.8 – 3.9 mmol/l)	266
[0.194]	Increased (5.1 – 7.5)	15
γ-GT (n = 459)	Normal (9 – 30 U/l)	389
(22, 9, 169 U/l) [0.000]	Increased (31 – 169 mmol/l)	70
ASAT (n = 458)	Normal (20 – 103 U/l)	377
(78, 45, 1070 U/l) [0.000]	Increased 104 – 1070 U/l)	81
Venous blood gas		
pH (n = 426)	Normal (7.41 – 7.45)	142
(7.9, 5.0, 10.0)	Decreased (7.10 – 7.40)	264
[0.000]	Increased (7.46 – 7.50)	20
pCO_2_ (n = 426)	Normal (35.0 – 45.0)	298
(40.7, 24.4, 72.6 mmHg)	Decreased (24.4 – 35.0)	54
[0.000]	Increased (45.1 – 72.6)	74
Bicarbonate (n = 426)	Normal (20.0 – 30.0)	362
(23.6, 14.3, 40.6 mmol/l)	Decreased (14.3 – 20.0)	49
[0.000]	Increased (30.1 – 40.6)	15
Base excess (n = 426)	Normal (−2 to +2)	182
(0.20 ± 3.9 mmol/litre)	Decreased (−14.6 to −2.8)	116
[0.051]	Increased (2.1 to 16.1)	128

There was a higher than normal concentration of blood urea nitrogen in 40.7% and a higher than normal concentration of bilirubin in 32.2% of cattle.

Hypocalcaemia was present in 85.1%, hypokalemia in 58.3%, hyponatraemia in 41.6% and hypophosphataemia in 40.5% of cases.

The blood pH was less than 7.4 in 62.0% and greater than 7.45 in 4.7%. A negative base excess (BE < −2 mmol/l) was recorded in 27.2% and a positive base excess (BE > + 2 mmol/l) in 30%.

The urine pH was normal in 202 of 417 (48.4%) cattle, increased (pH > 8.0) in 107 and decreased (pH < 7.0) in 108. Ketone bodies were found in the urine of 53 (12.7%) cattle (approximate values, 10 mg/dl in 29, 50 mg/dl in 17, 150 mg/dl in 7).

The chloride concentration of ruminal fluid was greater than normal in 70 of 404 (17.3%) cattle with values of 26 to 45 mmol/l.

### Preoperative diagnosis

Based on the results of rectal palpation, caecal dilatation was diagnosed in 291 (63.2%) cattle, caecal dilatation and retroflexion in 94 (20.4%) and caecal dilatation and torsion in 20 (4.3%). Caecal dilatation could not be diagnosed via rectal palpation in 56 (12.1%) cases; ultrasonography, laparotomy or postmortem examination was required to make a diagnosis in those cattle.

### Treatment (overview)

**Figure 1:**
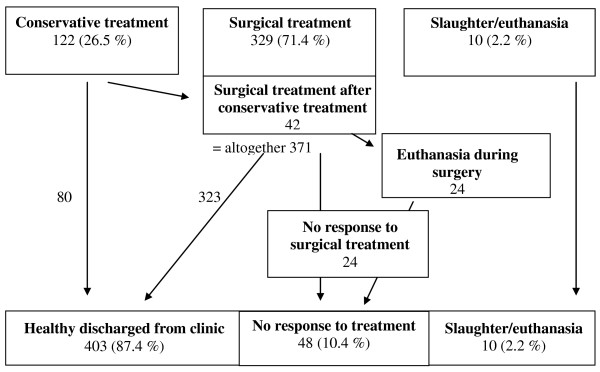
**Overview of the treatment.** Overview of the treatment of 461 cattle with caecal dilatation

Of the 461 patients, 122 received conservative treatment initially and 329 were operated (Figure 1). Ten cattle were euthanased or slaughtered after the initial physical examination. Forty-two of the cattle treated conservatively required surgery because of a lack of response after one to two days of conservative therapy. The total number of cattle operated was 371 (80.5%). Twenty-four cattle were euthanased or slaughtered intraoperatively because of a grave prognosis. Twenty-four other cattle were euthanased or slaughtered shortly after one or more operations because of deteriorating condition; of the 24 cases, six were operated twice and three thrice. Of the 461 patients, 403 (87.4%) were healed and discharged from the clinic and 58 (12.6%) were euthanased or slaughtered.

### Type of operation and measures after recurrence

During initial surgery, the caecum was emptied in 265 cattle and partially amputated in 41 (Table 3). In 48 (10.4%) patients, recurrence of caecal dilatation occurred 2 to 29 days (med. 2.5 days) postoperatively; 45 underwent a second operation and three of these had a third operation. In six cases, the caecum was re-emptied and in 32, the caecum was amputated. One patient was reoperated because of peritonitis caused by adhesions. Five cattle had to be euthanased during the second operation because of serious complications, which included caecal rupture, suture line dehiscence, intussusception of the colon, rupture of the colon wall and adhesions. One patient died during the second operation.

**Table 3:** Surgical treatment and outcome of 371 cattle with caecal dilatation

Variable	One operation	Two operations	Three operations
Number of patients	323 (100%)	45 (100%)	3 (100%)
Caecotomy to empty caecum	265 (82.0%)	6 (13.3%)	1 (33.3%)
Amputation	41 (12.7%)	32 (71.2%)	1 (33.3%)
Treatment of peritonitis	–	1 (2.2%)	–
Euthanased/died during surgery	17 (5.3 %)	6 (13.3 %)	1 (33.3 %)
Cured/discharged from clinic	290 (89.8 %)	33 (68.6 %)	0 (0 %)

A total of 33 of the 45 cattle that underwent two operations were healthy at the time of discharge from the clinic. Recurrence of caecal dilatation occurred one day after the second operation in one patient and three days after the second operation in two others, and all were operated a third time. One was euthanased during the third operation because of suture dehiscence and putrid peritonitis and the other two were euthanased one day postoperatively because of deterioration in general condition.

### Outcome

In the conservatively-treated cattle, normalisation of the general condition and demeanour occurred one to four days (median 1.0 day) after the start of therapy, and appetite and faecal output returned to normal one to three days (median 1.0 day) and one to five days (median 1.0 day) after treatment, respectively. Those cattle were discharged from the clinic three to 19 days (median 5.0 days) after admission. In operated cattle, it took one to seven days for the general condition and demeanour (median 1.0 day; difference to conservatively-treated cattle P = 0.99), appetite (median 1.0 day; difference to conservatively-treated cattle P = 0.39) and faecal output (median 2.0 days; difference to conservatively-treated cattle P = 0.25) to return to normal. Operated cattle were discharged from the clinic four to 29 days (median 6.0 days; difference to conservatively-treated cattle P = 0.77) after admission. Significantly (P < 0.01) more time was required for normalisation of the general condition and demeanour (1.7 versus 1.4 days) and faecal output (1.9 versus 1.4 days) in operated cattle than in conservatively-treated cattle.

### Comparison of cows with successful and unsuccessful outcomes

The rectal temperature at the initial examination was significantly higher in the 400 cows that recovered (median, minimum, maximum = 38.8, 35.7, 40.4 °C) than in the 58 cows that died or were euthanased (38.5, 36.2, 39.9 °C) (P < 0.01). The heart rate was significantly lower in cows that recovered (72, 42, 124 bpm) than in cows that did not (80, 56, 160 bpm) (P < 0.01). Ruminal atony was more common in cows that had an unsuccessful outcome (16.9%) than in cows that recovered (5%).

The following haematological and biochemical variables were significantly different between cows that recovered and those that did not (P < 0.05): Total erythrocyte count (6.41, 2.05, 12.30 vs. 6.88, 4.99, 10.20 x 10^6^/μl), PCV (32.4 ± 4.39 vs. 35.6 ± 7.43%), total leukocyte count (9.2, 1.0, 35.1 vs. 11.2, 2.9, 24.5 x 10^3^/μl), the serum concentrations of urea nitrogen (6.0, 1.4, 33.3 vs. 6.8, 2.6, 23.5 mmol/l), sodium (144.9 ± 3.56 vs. 143.7 ± 4.39 mmol/l), chloride (102, 73, 119 vs. 97, 68, 111 mmol/l), magnesium (1.0 ± 0.20 vs. 1.1 ± 0.35 mmol/l) and inorganic phosphorus and blood pH (7.8, 5.0, 10.0 vs. 8.0, 6.0, 9.0).

## Discussion

Characteristic clinical signs of caecal dilatation described in standard textbooks [1,14] were seen in the patients of the present study. The general condition and demeanour were abnormal in 93.1% of cattle, and signs of colic, such as shifting weight in the hind limbs, kicking at the abdomen and sinking of the back, were observed in 32.1%. Ruminal motility was reduced in 78.3% of cases, and faecal output was decreased or absent in 82.4%. Similar findings were reported in other large studies [4,8,9,12]. Tests for reticular foreign bodies were positive in 20.4% of the cases, probably because of pain elicited by mechanical stress on the dilated caecum and associated mesentery. In a previous study [4], 58% of cattle with caecal dilatation had a positive reaction to reticular foreign body tests. Some authors [15-17] reported distension of the right flank as a sign of caecal dilatation. However, in the present study, only 51 cattle had a change in the shape of the abdomen and of those, 42 (9% of all patients) had distension of the right flank, which did not make this a reliable diagnostic sign. The rectal temperature was below normal in 25.2% of patients, which was likely attributable to circulatory centralisation. A positive swinging and/or percussion auscultation of the right abdominal wall was an important finding because it occurred in 82.6% of patients. Although this is suggestive of caecal dilatation, right abomasal displacement and ileus may produce a similar pinging noise on percussion auscultation. In one study [18], the most frequent cause of a positive swinging and percussion ausculatation test in the right flank was right abomasal displacement and torsion of the abomasum (and omasum). Other gastrointestinal disturbances such as caecal dilatation or ileus and non-gastrointestinal disorders occurred less often. The results of one study showed that caecal dilatation was the most common cause of positive swinging and/or percussion auscultation in the right flank of cattle [19]. In 87.9% of patients, caecal dilatation was diagnosed via rectal palpation, which was a more important diagnostic tool than swinging and/or percussion auscultation. Rectal palpation allowed an accurate diagnosis in the majority of cases, which is in agreement with another study [4].

Hypocalcaemia occurred in 85.1% of cases and was the most important laboratory finding, in agreement with a previous study [5]. However, it is not known whether the hypocalcaemia was a cause or a result of caecal dilatation. Caecal dilatation occurs more often in cattle that are not fed a mineral supplement [11], which indicates that calcium deficiency, most likely along with other factors, may cause caecal dilatation. Intestinal obstruction leads to increased myoelectrical activity of the intestine resulting in increased consumption of calcium [20,21]; thus it is conceivable that hypocalcaemia results from caecal dilatation. Hypokalaemia occurred in 58.3% of patients, which was likely due to decreased feed intake. The higher than normal blood urea nitrogen concentration in 40.7% of the cattle was most likely attributable to dehydration leading to prerenal azotaemia, because causes of renal and postrenal azotemia were ruled out. Seventy cases had an increased concentration of ruminal chloride, which has been attributed to duodenal compromise causing abomasal reflux in some cases with caecal dilatation [5,22].

A total of 122 (26.5%) cattle initially received conservative treatment. Of those, 42 did not respond to treatment and required surgery. Thus, only 17.4% of cases were cured with conservative treatment, and conservative treatment was successful in only 65.5% of cattle for which this treatment was first selected. An older study [6] reported that 10.8% of cattle with caecal dilatation were successfully treated conservatively. The results of the present study are in agreement with other reports in which conservative treatment lead to a cure rate of 19.7% of 71 cows [23] and 21.4% of 84 cows [9], respectively. Of the 371 patients that underwent surgical therapy, 323 (87.1%) were cured. In previous studies, cure rates of 77.1 [9], 90.4 [24] and 90.9% [6], respectively, were reported. In the present study as well as in similar studies [6,8,16], the reason for choosing surgical treatment was based primarily on the presence of colic, signs of cardiovascular compromise, no faecal output and torsion or retroflexion of the caecum. The recurrence rate after the first operation was 13.8%, which is in agreement with the results of other studies (10.0%) [9], 12.6% [6]). Emptying of the caecum is considered by some to be a faster, less expensive and easier procedure than caecal amputation [25]. However, a higher rate of recurrence should be expected if amputation is avoided. For this reason, owners of operated cattle must be informed of the risk of recurrence and instructed to optimize the ration as a prophylactic measure [26,27]. Although partial amputation of the dilated caecum is associated with a low risk of recurrence, it is a more demanding procedure and has a high rate of complications. For those reasons, it should be reserved for cases in which there is recurrence after caecotomy or ischaemia of the caecal wall, because the short- and long-term outcome of patients with caecal dilatation is not improved by partial amputation of the caecum. The only significant differences, although minor, between operated cattle and those treated conservatively were the number of days required for faecal output and general condition and demeanour to normalise. In both groups of cattle, appetite, faecal output and general condition and demeanour usually improved one day post treatment.

In summary, 403 (87.4%) cattle with caecal dilatation were healthy at the time of discharge from our clinic. Recovery rates of 73.8% [9], 85.7% [8] and 91.0% [6] have been reported in other studies.

Although there were significant differences in rectal temperature, heart rate, ruminal motility and several haematological and biochemical variables between the 400 cows that recovered and the 58 that did not, these variables could not be used to determine the prognosis. This was because the variables had very similar means and large variations, which made them invalid as prognosticators. Instead, the clinician should consider all patient findings including clinical and laboratory results as well as the duration of the illness before making a prognosis.

## Conclusions

Caecal dilatation can usually be diagnosed based on clinical findings and treated conservatively or surgically. Swinging and percussion auscultation as well as rectal examination are important diagnostic tools. Conservative treatment is not rewarding in cattle considered surgical candidates with suspected caecal torsion or retroflexion and surgery should not be delayed in these patients.

## Consent

Written informed consent was obtained from the patients for publication of this report and any accompanying images.

## Competing interests

The authors declare that they have no competing interests.

## Authors' contributions

UB initiated and planned the study, examined many of the cows and wrote the manuscript, CB analysed the records, CG and GKS examined many of the cows and supervised less-experienced veterinarians, EM and KN operated the cows and wrote the surgical part of this manuscript and MH did the statistical evaluation. All authors have read and approved the manuscript.

## Sources of findings

This study was financed by the University of Zurich, Switzerland.
